# Diagnosing Mucopolysaccharidosis type IV a by the fluorometric assay of N-Acetylgalactosamine-6-sulfate sulfatase activity

**DOI:** 10.1186/s40200-017-0319-1

**Published:** 2017-09-08

**Authors:** Sedigheh Shams, Maliheh Barazandeh Tehrani, Gabriel Civallero, Koosha Minookherad, Roberto Giugliani, Aria Setoodeh, Mohammad Taghi Haghi Ashtiani

**Affiliations:** 1 Children’s Medical Center, Pediatrics Center of Excellence, Tehran University of Medical Sciences, Tehran, Iran; 2 Department of Pathology, Tehran University of Medical Sciences, Tehran, Iran; 3 Pediatric Urology Research Center, Tehran University of Medical Sciences, Tehran, Iran; 4 Department of Medicinal Chemistry, Faculty of Pharmacy and Pharmaceutical Sciences Research Center, Tehran University of Medical Sciences, Tehran, Iran; 5 Medical Genetics Service, Hospital de Clinicas de Porto Alegre, Porto Alegre, Brazil; 6 Department of Genetics, Federal University of Rio Grande do Sul, Porto Alegre, Brazil; 7 Division of Pediatrics Endocrinology; Children’s Medical Center, Pediatrics Center of Excellence, Tehran University of Medical Sciences, Tehran, Iran

**Keywords:** MPS IV A, Mucopolysaccharidosis, Morquio A, Enzyme assay, GALNS deficiency

## Abstract

**Background:**

Mucopolysaccharidosis type IVA, also known as Morquio A or MPS IV A, is an autosomal recessive disease caused by the deficiency of the lysosomal enzyme N-acetylgalactosamine-6-sulfate sulfatase (GALNS). The loss of GALNS activity leads to the impaired breakdown of glycosaminoglycans (GAGs) keratan sulfate and chondroitin-6-sulfate. The accumulation of GAGs results in multiple organ damage. The accurate and early diagnosis of this disorder helps enhance the effectiveness of the treatment. The present study uses a pre-designed protocol for testing GALNS activity in the leukocytes of Iranian patients with MPS IV A and their parents and compares it with healthy controls.

**Methods:**

Patients with MPS IVA previously diagnosed through the measurement of enzyme activity or genetic analysis entered the study. Leukocytes were obtained from the heparinized blood of the participants. The GALNS activity was measured by a fluorometric method using 4-methylumbelliferyl-β-D-galactoside-6-sulfate (4MU-G6S) as the substrate and proper buffer solutions and calibrators.

**Results:**

The GALNS activity (nmol/17 h/mg protein) was reported as 0–7.4 in the MPSIV A patients, as 19.85–93.7 in their parents and as 38.4–164 in the healthy controls. Statistically significant differences were observed between the three groups in terms of enzyme activity. There were no significant differences in enzyme activity by age. The female subjects in both the patient and parents groups showed lower enzyme activity compared to the male subjects.

**Conclusion:**

The fluorometric method was validated for the measurement of GALNS activity in leukocyte samples and identifying Iranian patients with MPS IV A.

## Background

Mucopolysaccharidosis type IV-A (OMIM #253000) or Morquio syndrome type A or simply MPS IV A is an autosomal recessive disease caused by the deficiency of the lysosomal enzyme N-acetylgalactosamine-6-sulfate sulfatase (GALNS). Due to the deficiency of this enzyme, glycosaminoglycans (GAGs) such as keratan sulfate (KS) and chondroitin-6-sulfate (CS) are not decomposed and instead accumulate in different organs. The accumulation of GAGs in different organs causes many clinical manifestations, including skeletal, cardiopulmonary and visual disorders. Skeletal disorder symptoms begin mainly with short stature and dysostosis multiplex. The height does not exceed 113–123 cm in patients with MPS IV A and the disease is often diagnosed with kyphosis, pigeon chest deformity, kyphoscoliosis and facial deformities. Unlike other mucopolysaccharidosis, in which the patients suffer from joint stiffness, patients with this disease have joint hypermobility due to ligamentous laxity. This condition makes walking difficult for the patients and has significant adverse effects on their quality of life. Dental problems are also characteristic of this disease. Patients with MPS IV A are cognitively and developmentally normal. Although patients with MPS IV-B (OMIM#253010) are phenotypically similar to those with MPS IV A and excrete KS as well, their disorder is caused by the deficiency of beta-galactosidase [1–5]. To date, over 220 mutations in the GALNS gene have been reported. Multiple mutations lead to the emergence of different phenotypes of this disease that makes the clinical diagnosis of the condition difficult, especially since many of its clinical symptoms are also observed in others types of MPS. In 2013, a group of scientists from different countries developed a guideline for the diagnosis of the disease so that physicians and laboratory directors can make an accurate and early diagnosis of the disease [2, 6, 7].

The screening methods used for the diagnosis of this disease include the analysis of GAGs in urine and the measurement of the enzyme activity in dried blood spots (DBS). Detecting the type of MPS excretion contributes significantly to the diagnosis of the disease, although it is not a very specific criterion. The absence of GAGs from the urine does not exclude the possibility of MPS IV A [8–13].

The final diagnosis is made by measuring enzyme activity in the leukocytes (whole blood) or fibroblasts (skin) and molecular tests can also confirm the diagnosis. In the case of a reduced enzyme activity in the fibroblast sample, Multiple Sulfatase Deficiency (MSD) should be ruled out.

Until recently, patients with this disease received only supportive care; however, in February 2014, the FDA approved the use of enzyme replacement therapy (ERT) with Elosulfase for treating this disease [14–16]. Given that the disease is progressive and irreversible, its early diagnosis and treatment can help improve the effectiveness of the treatment. Furthermore, accessing diagnostic methods for individuals with a family history of the disease and suspected patients can also be helpful. There are no precise statistics on the prevalence of MPS in Iran; however, it is likely more prevalent than the estimates due to the abundance of consanguineous marriages in the country. The present study was conducted to determine the activity of GALNS in healthy individuals and patients with MPS IV A and their parents for the first time in Iran so as to provide a more affordable method of diagnosis and to ease the access to the biochemical identification of the disease and enable its prenatal diagnosis across the entire country. To accomplish these goals, a fluorometric method previously presented was used in this study with slight changes [17].

## Methods

### Study subjects

The study subjects consisted of 12 patients with a Morquio A (MPS IV A) previously diagnosed with this disease through the measurement of enzyme activity or genetic studies performed outside Iran. A total of 11 parents of the patients also participated in the study, and two families had more than one child with this disease.

The control group consisted of a total of 20 healthy randomly-selected subjects who entered the study upon providing their informed consent. The patients and their parents also provided their written consent for participation in the study. This descriptive study was conducted at Children’s Medical Center, a referral teaching hospital in Tehran, in 2016. The study was approved by the Ethics Committee of Tehran University of Medical Sciences. The researchers complied with the ethical principles of the Declaration of Helsinki, including the confidentiality of the data, giving the physician in charge access to the lab test results if needed and not charging the patients for the study procedures.

### Reagents and solutions

The reagents used included dextran, albumin, sodium acetate, sodium azide, β-galactosidase galactohydrolase and 4- methylumbelliferone (calibrator) and were purchased from Sigma Co. The other reagents used were from Merck Co. The protein measurement kit was made in Iran by Pars Azmoon. The 4-Methylumbelliferyl β-D-Galactopyranoside-6-sulfate substrate was purchased from Sigma Co. and from Toronto Research Chemicals (TRC) in Toronto, Canada.

### Enzyme activity measurement

Five milliliters (5 ml) of heparinized blood was drawn from the participants and their leukocytes were immediately separated after adding an equal volume of 6% dextran (Sigma D-4876, Mw 150.000) using the method proposed by Diggelen and Skoog et al. [17, 18]. The separated leukocytes were sonicated with 500 μl of distilled water and then centrifuged so as to measure their protein concentration with a pyrogallol red-molybdate complex using a Vitalab Selectra E, Vital Scientific N. V. autoanalyzer. The samples were kept at −20 °C until the tests began. The enzyme activity was measured using the methods proposed by Diggelen and Camelier [17, 19] with slight changes. To summarize the measurements, 20 μl of the sample diluted with albumin solution (0.2% BSA and 0.2% Sodium Azide) and 40 μl of the substrate were incubated in a shaking bath at 37 °C for 17 h. In the second step, 10 μl of phosphate buffer solution and 20 μl of β-galactosidase galactohydrolase were added and the incubation continued for 2 h. Exogenous galactosidase was added to inhibit the effect of a potential galactosidase deficiency on the test results. The reaction was then stopped by adding carbonate buffer, centrifuged at 3000 rpm and the fluorescence was measured. The blank solution was made with the addition of the albumin solution instead of the sample (Table 1). The enzyme activity was measured using a calibration curve at different concentrations of 4-methylumbelliferone and reported in nmol/17 h/mg protein. The fluorescence of the samples and the calibrator was measured using a fluorometer (Model 450, Sequoia-Turner) at the excitation and emission wavelengths of 390 nm and 500 nm, respectively.

**Table 1:** The assay procedure for N-acetylgalactosamine-6-sulfate sulfatase (GALNS) activity in leukocytes

	Sample	Blank
Diluted Leukocyte (10 μg protein in BSA)	20 μl	–
Substrate^a^	40 μL	40 μL
BSA	–	20 μl
17 h at 37 °C with gentle shaking		
Phosphate Buffer^b^	10 μL	10 μL
Galactosidase Galactohydrolase 10 U/ml in BSA 0.2%	20 μl	20 μl
2 h at 37 °C with gentle shaking		
Stop Buffer^c^	800 μL	800 μL

^a^10 mM 4-MU-βGal-6S in 0.1 M Sodium Acetate, 0.1 M Acetic Acid with 0.02% Sodium Azide and 5 mM Lead Acetate Buffer, pH 4.3

^b^0.9 M Dibasic and Monobasic Sodium Phosphate Buffer with 0.02% Sodium Azide

^c^0.5 M Carbonate Buffer with 0.025% triton X-100, pH 10.7

The inter-assay variation coefficient was calculated through measuring the enzyme activity of the three samples on five different days. The intra-assay CV was calculated through measuring the enzyme activity of the three samples three times and in the same run.

### Statistical data analysis

The data obtained were analyzed in SPSS-17. The Kolmogorov-Smirnov test was used to examine the normal distribution of the data. The descriptive data were reported as mean ± standard deviation and minimum and maximum values for the quantitative data and as percentage for the qualitative variables.

The ANOVA and Bonferroni’s post-hoc tests were used to find statistical differences between the groups. The correlation coefficient was used to find correlations, if any, between the quantitative variables. *P* < 0.05 was considered as the level of statistical significance.

## Results

The present study was conducted on a total of 43 individuals, including 12 patients aged 1.5–24, 11 of their parents aged 25–58, and 20 seemingly healthy individuals aged 1.5–54 years old. The enzyme activity of the leukocytes was measured in the participants. The mean enzyme activity differed significantly between the three groups and was significantly lower in the group of patients compared to the other two groups and was also 50% less in the group of parents compared to the control group. The patients’ results did not overlap with the results of the other two groups in any aspect; however, an overlap was observed between the control group and the group of parents. Table 2 presents the distribution of the participants in the groups, their demographic data and the measured parameters.

**Table 2:** Demographic data and GALNS^a^ activity in the study population

	Patients	Parents	Controls	*P*-value^**^
	*n* = 12	*n* = 11	*n* = 20	
Female/Male	4/8	7/4	14/6	0.115
Age (Years) Mean ± SD	9.8 ± 7.1 (1.5–27)	37.4 ± 9.3 (25–58)	24.3 ± 15.1 (1.5–54)	<0.001
GALNS^a^ Activity Mean ± SD (nmol/17 h/mg protein)	1.5 ± 2.4 (0–7.4)	45.8 ± 25.7 (19.85–93.7)	78.7 ± 35.3 (38.4–164)	<0.001

^a^N-Acetyl Galactosamine-6- Sulfate sulfatase

**The mean difference is significant at the 0.05 level according to the ANOVA and Bonferroni’s post-hoc test

The enzyme activity was examined in and between the three groups by gender. The mean enzyme activity was lower in women than in men in the group of patients and parents, but higher in women than in men in the control group; however, the difference was not statistically significant (Table 3).

**Table 3:** Enzyme activity (nmol/17 h/mg protein) in the study groups by gender

	Patients^a^ Mean ± SD (Min-Max)	Parents^b^ Mean ± SD (Min-Max)	Controls^c^ Mean ± SD **(**Min-Max)	*P*-value^*^
Female	0.72 ± 1.45 (0–2.9)	39.6 ± 26.9 (19.85–93.7)	80.26 ± 35.9 (39.3–164)	a vs b: 0.173 a vs c: <0.001 b vs c: 0.029
Male	1.9 ± 2.7 (0–7.4)	56.7 ± 22.5 (36.4–88.3)	75.0 ± 36.8 (38.4–143)	a vs b: 0.005 a vs c: <0.001 b vs c: 0.743
*P*-value	0.287	0.314	0.770	

*The mean difference is significant at the 0.05 level according to the ANOVA and Bonferroni’s post-hoc test﻿

^a^Patients, ^b^Parents, ^c^Controls

The comparison of the three groups showed that the mean enzyme activity differed significantly between the female patients and their mothers (carriers) and the female controls. The mean enzyme activity in the female carriers was 50% less than that in the female controls.

The difference between the mean enzyme activity of the male carriers and the male controls was not statistically significant (*P* = 0.743); however, the male patients differed significantly in this regard from both the fathers of the patients and the male controls. The mean enzyme activity in the group of fathers was 75% as that in the male controls. No correlations were observed between the mean enzyme activity and age in the study groups.

The mean coefficient of variation was 7.3% for the assays in the same day and 22% for the assays in different days.

## Discussion

The present study is the first in Iran to have examined the GALNS activity in healthy individuals and patients with MPS IVA and their parents. A fluorogenic substrate and leukocytes separated from heparinized blood were used for this purpose. The results enabled the accurate differentiation of patients from healthy subjects and carriers.

Diggelen et al. synthesized the fluorogenic substrate 4-MU-Gal-6-S for the first time in 1990 and used it to diagnose enzyme deficiency in patients with Morquio A [17]. They compared this substrate with a radioactive substrate previously used for measuring this enzyme and proved the substrate to be suitable for diagnosing the disease. This method did not require the substrate to be separated and was more sensitive and economical than the radioactive substrate method. The fluorogenic substrate used in the present study also revealed a significant difference between the three groups.

In the study by Diggelen, the mean enzyme activity of the parents was 50% as that of the healthy population, which is consistent with the present study, which found that the mean enzyme activity of the parents was 50% as that of the controls. In the present study, the enzyme activity was lower than the control’s mean activity in more than 70% of the parents and lower than the minimum in 45% of them.

The mean enzyme activity was lower in women than in men in the group of patients and their parents; in the control group, however, it was slightly higher in women than in men, although the difference was not statistically significant. No gender-based comparisons were performed in Diggelen’s study and the other reports. The enzyme activity may vary with the particular mutation of GALNS, and patients differ significantly in terms of clinical symptoms and age at the onset of symptoms by their type of mutation [20]. Only a few of the patients and none of the parents in this study had undergone genetic testing. Explaining the differences in enzyme activity and its relationship to clinical symptoms requires molecular tests. Due to the small sample size of the study and the lack of access to the different mutations of GALNS, no definite conclusions can be made about the reduced enzyme activity in the mothers compared to the fathers in the carrier group.

No correlations were observed in this study between enzyme activity and age. Previous studies have not examined enzyme activity by age.

The mean inter assay coefficient of variation was 22% on different days which is reportedly higher than in previous studies [19, 21]. The coefficient of variation can be reduced if identical aliquots of the samples and substrates are used on different days.

The present study used two types of substrate –one made by Sigma and another one by TRC—, and found the latter to yield more favorable results (the details of these findings are not presented). These results are in line with those obtained by Ullal et al., who compared four substrates in their study and found the TRC substrate to be the most efficient [21]. A highly influential factor in this experiment was the stage of leukocyte separation and enzyme extraction. Although the level of enzyme activity is reported in mg of protein, samples should be drawn again if the separated cells are not enough and the protein concentration in the samples is less than 50 mg/dl. It is necessary to avoid melting the sample and substrate again. In studies conducted by Ullal and Camelier, whose method was adopted in the present study, different sample and substrate storage conditions and the specificity of the method were also examined by testing patients with other lysosomal disorders [19, 21]. No such tests were repeated in the present study due to the high costs of the substrate and the high volume of blood required. Similar to any other sulfatase, finding a deficient activity requires the measurement of other sulfatases to exclude the possibility of multiple sulfatase deficiency [2].

With the emergence of new treatments, the accurate and early diagnosis of MPS IV-A becomes necessary. Camelier et al. adapted Diggelen’s method to measure the enzyme activity in DBS [19]. This procedure, later improved on by Ullal et al. [21], not only facilitates the collection and delivery of samples to the referential laboratory for testing, but also make this procedure more suitable for the neonatal screening of MPS IV A. Recently, a study conducted by Cozma et al., measured GALNS activity on a DBS using a fluorogenic substrate and measured the reaction product by LC/MS-MS [22]. Extending the results of this study to newborns can be a potentially effective tool for neonatal screening.

## Conclusion

A GALNS measurement method was validated in this study at a university referral center in Iran for the first time to help enable the diagnosis of patients with MPS IV A and determine the values of enzyme activity in healthy subjects in Iran. A study with a larger sample size is required to determine the reference interval for GALNS activity in healthy Iranian subjects. More studies with larger sample sizes are required on the effectiveness of this enzyme assay as a method for identifying carriers.
